# Nutritional benefit of remnant gastric preservation in patients with esophageal cancer undergoing radical esophagectomy and ileo-colon interposition

**DOI:** 10.1186/s12893-022-01704-x

**Published:** 2022-07-02

**Authors:** Junya Kitadani, Toshiyasu Ojima, Keiji Hayata, Taro Goda, Akihiro Takeuchi, Masahiro Katsuda, Shinta Tominaga, Naoki Fukuda, Tomoki Nakai, Shotaro Nagano, Hiroki Yamaue

**Affiliations:** Second Department of Surgery, School of Medicine, Wakayama Medical University, 811-1 Kimiidera, Wakayama, 641-8510 Japan

**Keywords:** Esophageal cancer, Esophagectomy, Ileo-colon interposition

## Abstract

**Background:**

This retrospective study aimed to investigate the short-term surgical outcomes and nutritional status of ileo-colon interposition in patients with esophageal cancer who could not undergo gastric tube reconstruction.

**Methods:**

Sixty-four patients underwent subtotal esophagectomy with reconstruction using ileo-colon interposition for esophageal cancer at the Wakayama Medical University Hospital between January 2001 and July 2020. Using propensity scores to strictly balance the significant variables, we compared treatment outcomes.

**Results:**

Before matching, 18 patients had cologastrostomy and 46 patients had colojejunostomy. After matching, we enrolled 34 patients (n = 17 in cologastrostomy group, n = 17 in colojejunostomy group). Median operation time in the cologastrostomy group was significantly shorter than that in the colojejunostomy group (499 min vs. 586 min; *P* = 0.013). Perforation of the colon graft was observed in three patients (7%) and colon graft necrosis was observed in one patient (2%) in the gastrojejunostomy group. Median body weight change 1 year after surgery in the cologastrostomy group was significantly less than that of the colojejunostomy group (92.9% vs. 88.5%; *P* = 0.038). Further, median serum total protein level 1 year after surgery in the cologastrostomy group was significantly higher than that of the colojejunostomy group (7.0 g/dL vs. 6.6 g/dL, *P* = 0.030).

**Conclusions:**

Subtotal esophagectomy with reconstruction using ileo-colon interposition is a safe and feasible procedure for the patients with esophageal cancer in whom gastric tubes cannot be used. Cologastrostomy with preservation of the remnant stomach had benefits in the surgical outcomes and the postoperative nutritional status.

**Supplementary Information:**

The online version contains supplementary material available at 10.1186/s12893-022-01704-x.

## Background

Esophageal cancer is the sixth leading cause of cancer-related death for men and the ninth for women worldwide [1]. Although esophagectomy with a two- or three-field lymph node dissection is still considered to be a potentially curative treatment, it is highly invasive and there is a high rate of morbidity, despite improvements in surgical technique and postoperative management [2–5]. Gastric tubes are generally used for the reconstruction after esophagectomy; they provide abundant blood flow and can be safely pulled-up to the neck [6, 7]. However, in cases with a previous history of gastrectomy, or with synchronous gastric cancer, gastric conduit cancer, or with loss of a gastric tube, instead of the stomach reconstruction, colon interposition or pedicled jejunal flap reconstruction with microvascular anastomosis (MVA) are performed [8–23]. In our institute, ileo-colon interposition is the first choice when it is not possible to perform gastric tube reconstruction. Advantages of ileo-colon interposition are that the Bauhin valve prevents regurgitation, there is a reservoir-like capacity in the cecum, and the closeness of the diameter of the terminal ileum and esophagus [24]. Meanwhile, disadvantages include great variety in mesenteric blood vessels, which may be a cause of ischemia, and it being a comparatively more complicated procedure with multiple anastomoses [22]. There is currently no consensus as to whether MVA should be routinely performed. In previous reports, colon interposition with MVA was not particularly less likely to result in anastomotic leakage [15, 19]. We have therefore adopted ileo-colon interposition without MVA. Series of subtotal esophagectomy with reconstruction using ileo-colon interposition without MVA for esophageal cancer have not been widely reported [10, 14, 16, 25]. This retrospective study therefore aims to investigate the short-term surgical outcomes of ileo-colon interposition in patients with esophageal cancer who cannot undergo gastric tube reconstruction. In particular, we highlight the effect of cologastrostomy on nutritional status when the residual stomach is preserved.

## Materials and methods

### Patients

This retrospective cohort study was conducted at the Wakayama Medical University Hospital (WMUH), Wakayama, Japan. This study was in agreement with the guidelines of the institutional ethics committee (approval number 3291) and was conducted in accordance with the Declaration of Helsinki. Sixty-four patients underwent subtotal esophagectomy with reconstruction using ileo-colon interposition for esophageal cancer at WMUH between January 2001 and July 2020. Clinicopathologic factors were evaluated retrospectively based on hospital records including on age, sex, history and type of previous gastrectomy, and on surgical factors including operative time and blood loss. Branches of the superior mesenteric artery including the right colic artery and the ileocolic artery (ICA) were evaluated using dynamic computed tomography (CT). Clinical and pathological stages were determined according to the TNM classification (UICC 8th edition) [26]. The severity of the postoperative complications after operation was estimated according to Clavien–Dindo classification [27]. The frequency of complications was examined, with January 2001 to December 2010 regarded as the early phase, and January 2011 to July 2020 regarded as the late phase.

### Surgical procedures

**Fig. 1:**
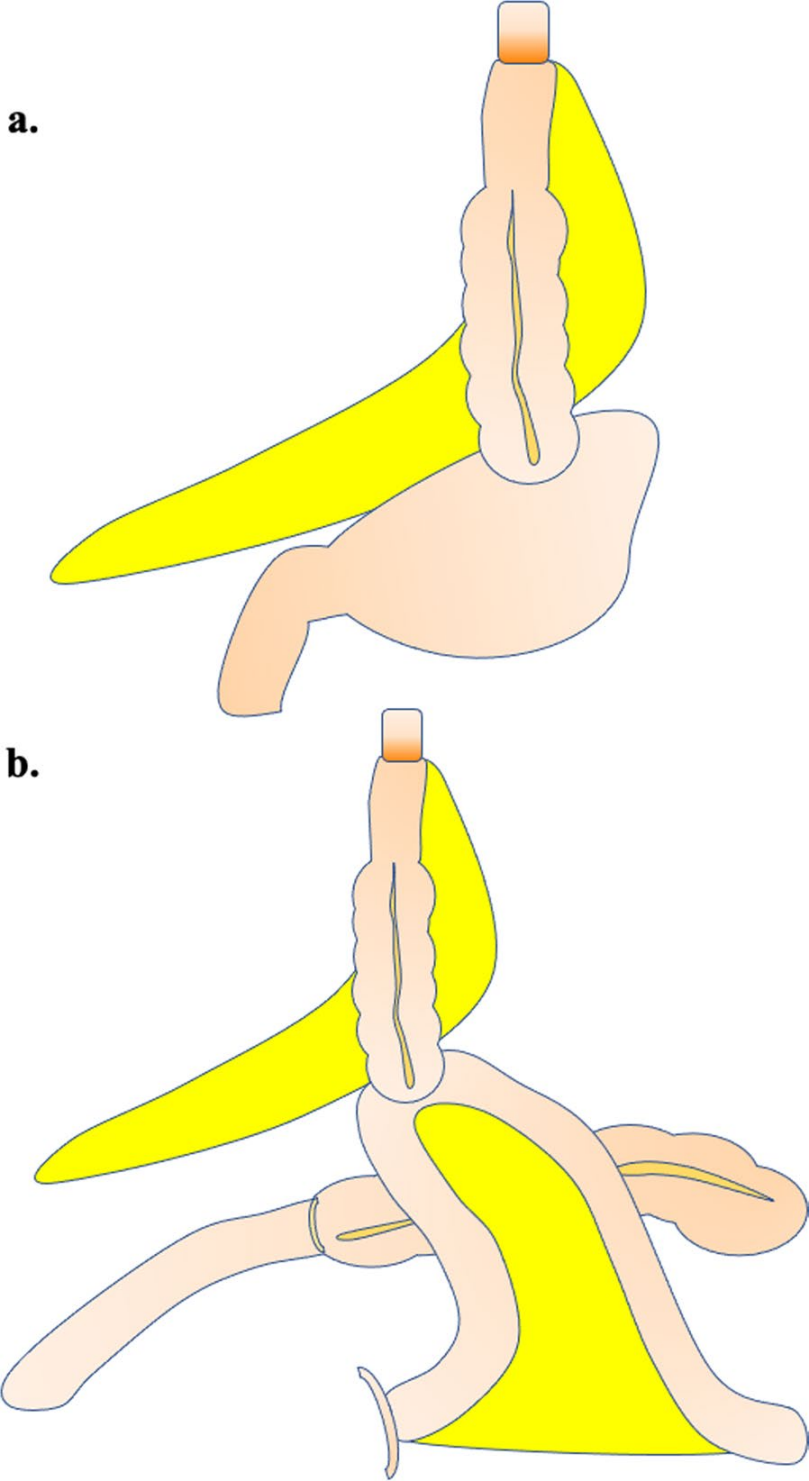
A schematic illustration of the ileo-colon interposition without MVA through the retrosternal route. **a** Anastomosis of cervical esophagus and ileum at the neck is performed, and gastrocolostomy is performed if the stomach can be preserved. **b** If the stomach cannot be preserved, ileocolostomy is performed

Until May 2010, 38 patients underwent open right transthoracic esophagectomy with two-field (total mediastinal, perigastric and coeliac regions) or three-field (adding supraclavicular and cervical paratracheal regions) lymph node dissections. The subsequent 26 patients underwent minimally-invasive thoracoscopic esophagectomy [5, 28]. The esophagus was transected with a linear stapler in the thoracic cavity depending on the location of the tumor. A midline incision was made in the upper abdomen to remove the section of the esophagus containing the tumor, and Kocher mobilization was performed to mobilize the duodenum and right colon from the retroperitoneum. The appendix was removed to prevent appendicitis. Colon interposition was performed under the following principles: (i) use the right hemicolon; (ii) preserve the right colic artery, as well as the right branch of the middle colic artery; (iii) dissect the ICA; (iv) trim the mesentery along the marginal vessels and transect the ileum with a linear stapler 20 cm from the ileum end; (v) lift the graft in the retrosternal route; (vi) do not perform supercharge or superdrainage unless the blood flow in the graft is clearly poor; (vii) anastomose cervical esophagus and ileum at the neck, remnant stomach and the colon (Fig. 1a), or the jejunum and colon (Fig. 1b); (viii) anastomose the anal-side transverse colon and ileum; (ix) insert a 16 Fr nasal tube into the colon graft and a 12 Fr feeding tube into the jejunum 30 cm from the Treitz ligament. Until 2012, all patients underwent total gastrectomy and colojejunostomy. After 2013, stomachs were intentionally preserved and patients underwent cologastrostomy.

### Nutritional status and follow-up

Food intake was usually started on the 8th day if there was no anastomotic leakage or severe dysphagia. Nutrition through a feeding jejunostomy or a feeding gastrostomy was continued for an average of 3 months until sufficient food intake was achieved. Follow-up was conducted every 3 months to measure body weight change, serum total protein and albumin values. This included systemic clinical examination and thoraco-abdominal dynamic CT scan and upper endoscopy every 6 months.

### Statistical analyses

All statistical analyses were carried out using JMP Pro 16.0 (SAS Institute Inc., Cary, NC, USA). Categorical variables were assessed using Chi square method. Continuous variables were evaluated using the Wilcoxon signed-rank test. Statistical significance was defined as *P *< 0.05. A propensity-matched analysis was conducted using the logistic regression model and covariates such as age, sex, comorbidity, tumor location, clinical stage, and presence or absence of neoadjuvant chemotherapy and chemoradiotherapy. One-to-one matching without replacement was completed using the nearest neighbor match on the logit of the propensity score with the caliper width set to 0.20 times the standard deviation of the logit of the propensity score.

## Results

### Patient characteristics

Sixty-four patients with esophageal cancer who underwent ileo-colon interposition were eligible for this retrospective study, of which 18 patients had cologastrostomy (Fig. 1a) and 46 patients had colojejunostomy (Fig. 1b). After matching, we enrolled 34 patients (n = 17 in cologastrostomy group, n = 17 in colojejunostomy group) (Additional file 1: Fig. S1). Cologastrostomy and colojejunostomy were performed via hand-sewn or functional end-to-end anastomosis. Table 1 shows comparison of patient characteristics between the cologastrostomy group and the colojejunostomy group. Age, gender, location of esophageal tumor, pathological stage, and whether or not the patient received neoadjuvant therapy were not significantly different between the groups. There was more history of total gastrectomy and synchronous disease in the colojejunostomy group than in the cologastrostomy group (*P* < 0.001).

**Table 1:** Comparison of patient characteristics between the cologastrostomy and colojejunostomy groups

Categories	Before matching		*P* value	After matching		*P* value
	Cologastrostomy group (n = 18)	Colojejunostomy group (n = 46)		Cologastrostomy group (n = 17)	Colojejunostomy group (n = 17)	
Age, median (quartiles), years	69.5 (67–74)	68 (64–72)	0.129	69 (67–75)	68 (65–73)	0.478
Gender			0.513			1.000
Male	18 (100%)	44 (96%)		17 (100%)	17 (100%)	
Female	0 (0%)	2 (4%)		0 (0%)	0 (0%)	
Comorbidity						
Cardiovascular diseases	2 (11%)	2 (4%)	0.313	2 (12%)	2 (12%)	1.000
COPD	2 (11%)	3 (7%)	0.615	2 (12%)	1 (6%)	1.000
Diabetes mellitus	2 (11%)	2 (4%)	0.313	1 (6%)	2 (12%)	1.000
Hypertension	2 (11%)	7 (15%)	1.000	2 (12%)	4 (24%)	0.656
Chronic kidney disease	0 (0%)	1 (2%)	1.000	0 (0%)	1 (6%)	1.000
Chronic liver disease	0 (0%)	6 (13%)	0.173	0 (0%)	1 (6%)	1.000
Histology			1.000			1.000
Squamous cell carcinoma	18 (100%)	46 (100%)		17 (100%)	17 (100%)	
Adenocarcinoma	0 (0%)	0 (0%)		0 (0%)	0 (0%)	
Location of esophageal tumor			0.254			0.822
Ut	1 (6%)	3 (6%)		1 (6%)	1 (6%)	
Mt	15 (83%)	27 (59%)		14 (82%)	12 (70%)	
Lt	2 (11%)	15 (33%)		2 (12%)	4 (24%)	
Ae	0 (0%)	1 (2%)		0 (0%)	0 (0%)	
pStage (TNM classification 8th edition)			0.121			0.916
0	1 (6%)	1 (2%)		1 (6%)	1 (6%)	
I	7 (39%)	9 (20%)		6 (35%)	8 (47%)	
II	6 (33%)	10 (22%)		6 (35%)	6 (35%)	
III	3 (16%)	21 (46%)		3 (18%)	1 (6%)	
IV	1 (6%)	5 (10%)		1 (6%)	1 (6%)	
Neoadjuvant therapy			0.876			0.688
Chemotherapy	4 (22%)	8 (17%)		5 (29%)	3 (18%)	
Chemoradiotherapy	1 (6%)	2 (5%)		0 (0%)	0 (0%)	
None	13 (72%)	36 (78%)		12 (71%)	14 (82%)	
History of gastrectomy			< 0.001			< 0.001
Total gastrectomy	0 (0%)	7 (15%)		0 (0%)	2 (12%)	
Distal gastrectomy	17 (94%)	17 (37%)		16 (94%)	5 (29%)	
Synchronous gastric cancer or ulcer	1 (6%)	22 (48%)		1 (6%)	10 (59%)	
Histological type of previous gastrectomy			0.307			0.083
Benign	6 (35%)	5 (21%)		6 (36%)	2 (29%)	
Malignant	11 (65%)	19 (79%)		10 (64%)	5 (71%)	

*COPD* chronic obstructive pulmonary disease, *Ut* upper thoracic esophagus, *Mt* middle thoracic esophagus, *Lt* lower thoracic esophagus, *Ae* abdominal esophagus

### Surgical outcomes and postoperative complications

Comparison of surgical outcomes between the cologastrostomy group and colojejunostomy group is shown in Table 2. The median operation time in the cologastrostomy group was significantly shorter than that in the colojejunostomy group (499 min vs. 586 min; *P* = 0.013). In all cases, ileo-colon interposition was performed through the retrosternal route. The right colic artery was present in 26 patients (40%) and was preserved in these cases. No patients underwent MVA. There were no differences in the rate of complications higher than Clavien–Dindo grade II or higher than Clavien–Dindo grade IIIa between the cologastrostomy and colojejunostomy groups. Early or late perforation of colon graft was observed in three patients (7%) and colon graft necrosis was observed in one patient (2%) in the gastrojejunostomy group. Two patients in the gastrojejunostomy group had graft loss, and both underwent second-stage jejunal reconstruction. There was no mortality in our consecutive series. The median length of postoperative hospital stay was not significantly different between the cologastrostomy and colojejunostomy groups (29 days vs. 29 days; *P* = 0.814). Table 3 shows comparison of postoperative complications between the early and the late phases. Overall morbidity of C–D grade ≥ 2 or C–D grade ≥ 3a were not significantly different between the early and the late phases. Anastomotic stenosis was significantly decreased in the case of late phase (*P* = 0.035).

**Table 2:** Comparison of surgical outcomes between the cologastrostomy and colojejunostomy groups

Categories	Before matching		*P* value	After matching		*P* value
	Cologastrostomy group (n = 18)	Colojejunostomy group (n = 46)		Cologastrostomy group (n = 17)	Colojejunostomy group (n = 17)	
Operation time, median (quartiles), min	494 (430–580)	600 (499–660)	0.001	499 (437–582)	586 (511–653)	0.013
Blood loss, median (quartiles), ml	209 (123–479)	485 (184–760)	0.037	225 (230–507)	550 (220–710)	0.067
Lymph node dissection			0.272			0.688
Two-field	13 (72%)	26 (57%)		12 (71%)	14 (82%)	
Three-field	5 (28%)	20 (43%)		5 (29%)	3 (18%)	
Reconstruction route			1.000			1.000
Retrosternal	18 (100%)	46 (100%)		17 (100%)	17 (100%)	
Posterior mediastinum	0 (0%)	0 (0%)		0 (0%)	0 (0%)	
Percutaneous	0 (0%)	0 (0%)		0 (0%)	0 (0%)	
Anastomosis method (neck)			0.068			0.017
Hand sewn anastomosis	2 (11%)	10 (22%)		2 (12%)	6 (35%)	
Circular stapler	7 (39%)	27 (59%)		7 (41%)	10 (59%)	
Functional end to end anastomosis	9 (50%)	9 (19%)		8 (47%)	1 (6%)	
Postoperative complications						
Overall morbidity (C–D grade ≥ 2)	10 (55%)	32 (69%)	0.289	10 (58%)	13 (76%)	0.465
Overall morbidity (C–D grade ≥ 3a)	7 (39%)	28 (61%)	0.112	7 (41%)	11 (65%)	0.303
Anastomotic leakage	2 (11%)	7 (15%)	1.000	2 (12%)	1 (6%)	1.000
Anastomotic stenosis	4 (22%)	19 (41%)	0.246	4 (24%)	9 (53%)	0.157
Respiratory complications	2 (11%)	9 (20%)	0.713	2 (12%)	5 (29%)	0.398
Recurrent nerve paralysis	3 (17%)	8 (17%)	1.000	3 (18%)	3 (18%)	1.000
Ileus	0 (0%)	2 (4%)	1.000	0 (0%)	1 (6%)	1.000
Reconstructive colon perforation	0 (0%)	3 (7%)	0.553	0 (0%)	1 (6%)	1.000
Reconstructive colon necrosis	0 (0%)	1 (2%)	1.000	0 (0%)	0 (0%)	1.000
Hernia of the graft	0 (0%)	1 (2%)	1.000	0 (0%)	0 (0%)	1.000
Graft loss	0 (0%)	2 (4%)	1.000	0 (0%)	0 (0%)	1.000
Arrhythmia	1 (6%)	3 (7%)	1.000	1 (6%)	1 (6%)	1.000
Chylothorax	1 (6%)	1 (2%)	0.487	1 (6%)	0 (0%)	1.000
Pneumothorax	1 (6%)	2 (4%)	1.000	1 (6%)	1 (6%)	1.000
Mortality	0 (0%)	0 (0%)	1.000	0 (0%)	0 (0%)	1.000
Post-operative hospital stays, median (quartiles), days	29 (24–36)	29 (25–59)	0.307	29 (25–36)	29 (24–47)	0.814

* C–D* Clavien–Dindo classification

**Table 3:** Comparison of postoperative complications between the early and late phases

Categories	Early phase (n = 35)	Late phase (n = 29)	*P* value
Overall morbidity (C–D grade ≥ 2)	23 (65%)	19 (65%)	1.000
Overall morbidity (C–D grade ≥ 3a)	19 (54%)	16 (55%)	1.000
Anastomotic leakage	3 (9%)	6 (20%)	0.278
Anastomotic stenosis	17 (48%)	6 (20%)	0.035
Respiratory complications	7 (20%)	4 (13%)	0.740
Graft loss	0 (0%)	2 (6%)	0.201

* C–D* Clavien–Dindo classification

### Nutritional comparison of the cologastrostomy and colojejunostomy groups

**Fig. 2:**
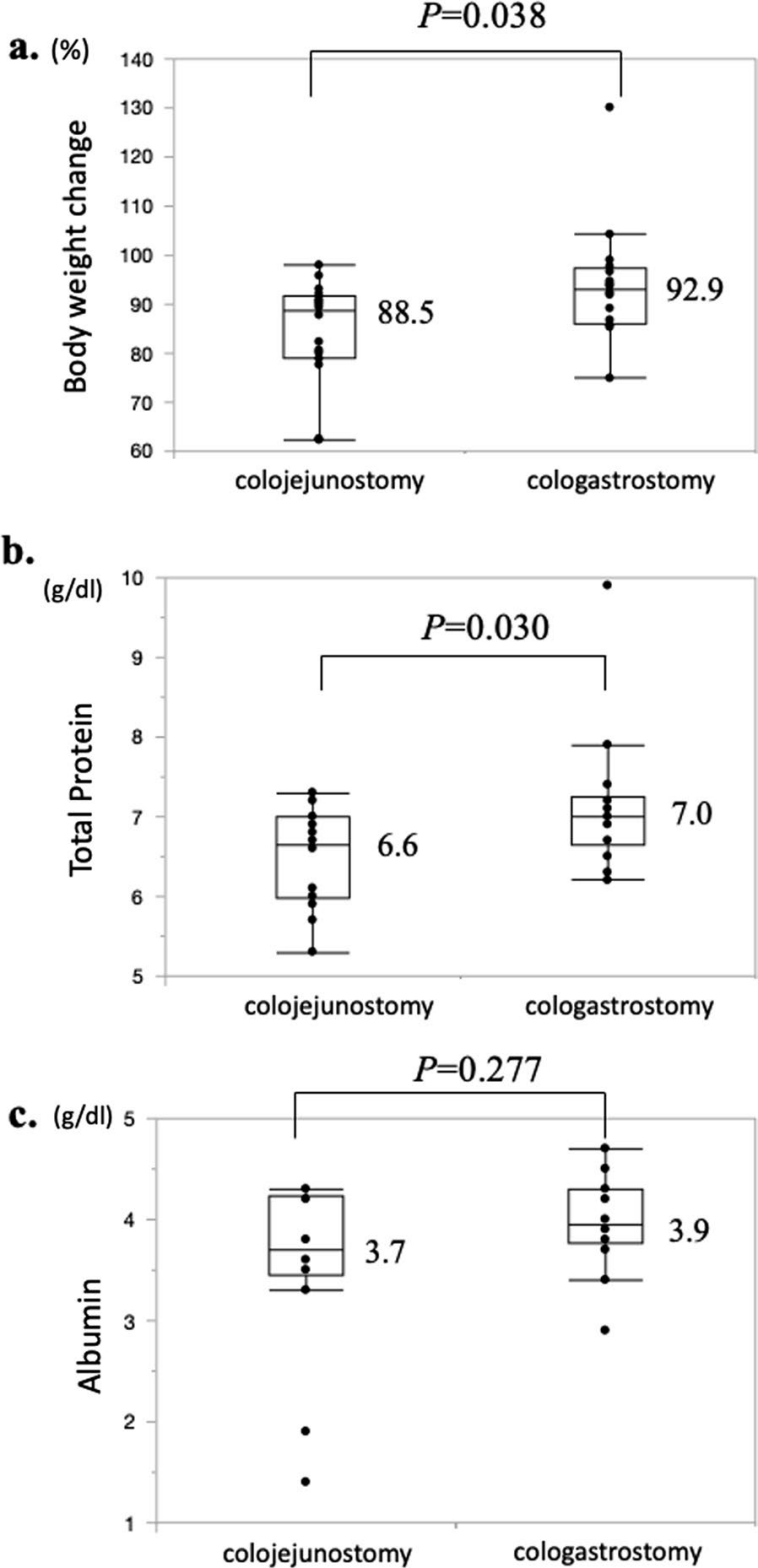
Nutritional comparison between the cologastrostomy group and the colojejunostomy group. **a** Median body weight change 1 year after surgery in the cologastrostomy group was significantly less than that of the colojejunostomy group (92.9% vs. 88.5%; *P* = 0.038). **b** Median serum total protein level 1 year after surgery in the cologastrostomy group was significantly higher than that of the colojejunostomy group (7.0 g/dL vs. 6.6 g/dL; *P* = 0.030). **c** Median serum albumin level 1 year after surgery in the cologastrostomy group was not significantly different from that of the colojejunostomy group (3.9 g/dL vs. 3.7 g/dL; *P* = 0.277)

Nutritional comparisons (body weight change, serum total protein level and serum albumin level) 1 year after esophagectomy and ileo-colon interposition between the cologastrostomy and colojejunostomy groups are shown in Fig. 2. Median body weight change 1 year after surgery in the cologastrostomy group was significantly less than that of the colojejunostomy group (92.9% vs. 88.5%; *P* = 0.038). Further, median serum total protein level 1 year after surgery in the cologastrostomy group was significantly higher than that of the colojejunostomy group (7.0 g/dL vs. 6.6 g/dL; *P* = 0.030).

## Discussion

This retrospective study shows that esophagectomy and ileo-colon interposition without MVA for patients with esophageal cancer was feasible and safe and without mortality. Cologastrostomy with preservation of the remnant stomach had benefits including short operation time and good postoperative nutritional status.

The rate of anastomotic leakage in our study was 14%, which was similar to the previously reported rate of anastomotic leakage in colon interposition with MVA [8, 12, 15, 19]. In our series, the retrosternal route was always selected so that the distance of the reconstruction route would be as short as possible. It also has better cosmetic results than the subcutaneous route. Our previous study showed postoperative anastomotic leakage to be an independent prognostic factor in ileo-colon interposition for patients with esophageal cancer [29]. Although there were no differences in the complications between the cologastrostomy group and the colojejunostomy group, there was no observation of very critical complications such as perforation or necrosis of the colon graft in the cologastrostomy group. This could be because preservation of the remnant stomach prevented abnormal expansion of colon graft. Although overall morbidity was not significantly different between the early and the late phases, anastomotic stenosis was significantly decreased in the case of late phase. This was thought to be because more functional end-to-end anastomosis using linear staplers was performed than hand-sewn sutures. If gastrectomy for gastric cancer had been performed, lymph node dissection of the lesser curvature side and supra-pancreatic area had already been completed. If the previous surgery is performed for a benign disease or a simultaneous ulcer, we suggest that only lymph node dissection should be added. In these cases, the remnant stomach can also be preserved while retaining blood flow of the gastrosplenic ligament. Advanced esophageal cancer that directly invades the remnant stomach requires total remnant gastrectomy. Also, if lymph node metastasis has infiltrated into the remnant stomach, total remnant gastrectomy is required. In this study there was no difference in recurrence or survival rates between the total gastrectomy and the remnant stomach-preservation groups (data not shown).

Nutritional status (body weight change, serum total protein level at 1 year after esophagectomy and ileo-colon interposition) in the cologastrostomy group was better than that in the gastrojejunostomy group. Among surgical procedures for gastric cancer, total gastrectomy has been shown to have disadvantages regarding nutritional status, including body weight loss [30, 31]. Furthermore, the remnant stomach may be associated with postoperative appetite stimulation because gut hormones such as ghrelin are secreted [30, 32].

In cases when gastric tube reconstruction could not performed, no clinical trials have yet ascertained whether colon interposition or pedicled jejunal flap reconstruction is better [8–10, 12–25, 33]. Pedicled jejunal flap reconstruction may be a promising procedure because fewer anastomoses are needed than in colon interposition. Creating a sufficient length of jejunal graft is sometimes difficult, however, especially in obese patients [22]. Surgical outcomes and quality of life of colon interposition and jejunal reconstruction will be examined in a future prospective cohort study based in multiple centers.

Several limitations associated with this study warrant mention. First, if the previous surgery was total gastrectomy, or if simultaneous gastric cancer requires total gastrectomy, it was inevitable that the stomach could not be preserved. Patients were allocated to the two groups according to the sequential nature of the surgery. Secondly, the number of patients with esophageal cancer in whom gastric tubes could not be used was relatively small, so only a small number of patients were ultimately eligible for this retrospective study, which was based in a single center. A prospective study will overcome the limitations of the retrospective design and selection bias.

## Conclusions

Subtotal esophagectomy with reconstruction using ileo-colon interposition is a safe and feasible procedure for patients with esophageal cancer in whom gastric tubes cannot be used. Cologastrostomy with preservation of the remnant stomach had benefits in surgical outcomes and postoperative nutritional status.

## Supplementary Information

**Additional file 1: Figure S1.** Flow chart showing patients included in the study.

## Data Availability

The datasets generated during and/or analyzed during the current study are not publicly available due to hospital regulations.

## Abbreviations

CT: Computed tomography
ICA: Ileocolic artery
MVA: Microvascular anastomosis
WMUH: Wakayama Medical University Hospital
