# Supplementary figures and images for: Nutritional benefit of remnant gastric preservation in patients with esophageal cancer undergoing radical esophagectomy and ileo-colon interposition
Source: BMC Surg. 2022 Jul 2;22:255. doi: 10.1186/s12893-022-01704-x (PMC9250726; doi:10.1186/s12893-022-01704-x)

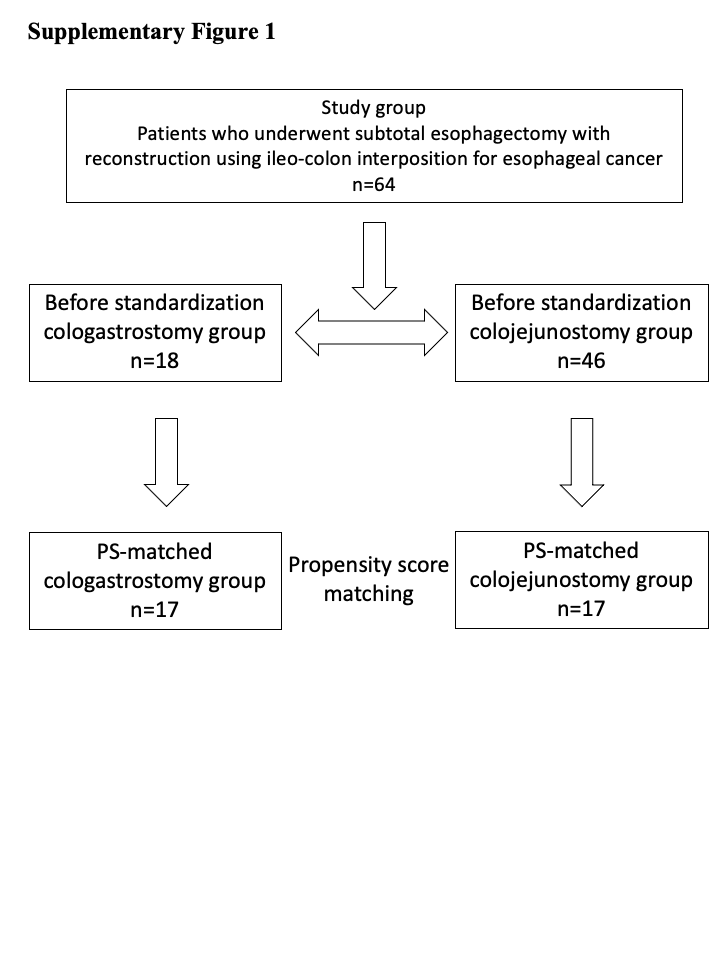

Supplement: Supplementary file 1 — Additional file 1: Figure S1. Flow chart showing patients included in the study. [file 12893_2022_1704_MOESM1_ESM.tiff]
